# Development of chimeric monoclonal antibodies as standardised positive controls in rickettsial serodiagnosis

**DOI:** 10.1038/s41598-026-55470-4

**Published:** 2026-05-29

**Authors:** Puntanat Tattiyapong, Suphasuta Khongpraphan, Stuart D. Blacksell, Jantana Wongsantichon

**Affiliations:** 1 Mahidol Oxford Tropical Medicine Research Unit, Faculty of Tropical Medicine, Mahidol University, Bangkok, 10400 Thailand; 2 Centre for Tropical Medicine and Global Health, Nuffield Department of Medicine, University of Oxford, Oxford, UK

**Keywords:** *Rickettsia typhi*, OmpB-typhi, Chimeric antibody, Monoclonal antibody, Serodiagnosis, Biological techniques, Biotechnology, Immunology, Microbiology

## Abstract

Rickettsial diseases present ongoing diagnostic challenges due to antigenic diversity, serological cross-reactivity, and the lack of reliable standardised control reagents. Current serodiagnostic assays frequently rely on convalescent human sera as positive controls, which are inherently variable, limited in supply, and poorly suited to assay standardisation and quality assurance. In this study, we developed and validated human-mouse chimeric monoclonal antibodies targeting the conserved outer membrane protein B (OmpB) of *Rickettsia typhi* as defined, renewable, positive-control calibrators for existing rickettsial serological platforms. Murine monoclonal antibodies were generated and engineered into chimeric IgG antibodies, which were expressed in HEK293T cells. The chimeric antibodies demonstrated high antigen specificity, bound native OmpB on the surface of *R. typhi*, and showed no detectable cross-reactivity within the tested antigen panel when evaluated using dot blot, immunofluorescence microscopy, and Luminex-based multiplex assays. Compared with patient-derived positive control sera, the chimeric antibodies demonstrated improved specificity and consistent performance within the evaluated assay conditions. These findings support the potential use of chimeric monoclonal antibodies as standardised positive controls for rickettsial serodiagnostics, with potential applications in assay validation, quality assurance, and inter-laboratory harmonisation.

**Supplementary Information:**

The online version contains supplementary material available at 10.1038/s41598-026-55470-4.

## Introduction

Rickettsial diseases are vector-borne zoonoses of major public health importance, particularly in endemic regions across Asia and the Pacific^1^. The causative agents, *Rickettsia* spp., are obligate intracellular bacteria responsible for diseases such as scrub typhus, murine typhus, and spotted fever group rickettsioses^2^. Despite their clinical significance, laboratory diagnosis remains challenging, and serology continues to underpin both clinical management and epidemiological investigations^3,4^.

Diagnosis remains largely dependent on serological assays, among which the indirect immunofluorescence assay (IFA) remains the gold standard^4^. However, IFA requires specialised facilities and trained personnel, making it less practical for routine or large-scale surveillance^4–7^. Alternative immunoassays, including enzyme-linked immunosorbent assays (ELISA) and Luminex-based multiplex assays, have gained attention for their higher throughput and potential for standardisation^7,8^. Luminex-based multiplex assays enable the simultaneous measurement of multiple analytes in a single run, making them more time-efficient and requiring smaller sample volumes compared to ELISA, which measures only a single analyte. This multiplex capability is particularly advantageous for evaluating antibody reactivity across multiple antigens^9^. Yet, their performance is often hindered by antigenic diversity, cross-reactivity^7^, and the lack of reliable positive control materials^10^.

Current controls are typically derived from convalescent human sera, which vary in antibody concentration, stability, and specificity^10,11^. These materials are inherently variable, limited in supply, and subject to degradation over time. Differences in antibody titre, specificity, and cross-reactivity introduce substantial batch-to-batch variability, undermining assay validation, quality assurance, and inter-laboratory comparability^12^. These limitations pose a significant barrier to assay harmonisation, regulatory evaluation, and the development of multiplex diagnostics for acute febrile illness^12^. To overcome these limitations, we sought to develop chimeric monoclonal antibodies that combine the antigen-binding specificity of murine mAbs with the constant regions of human IgG. These engineered antibodies can serve as standardised, renewable positive controls for serological assay validation and quality assurance in rickettsial disease diagnostics^13,14^.

In this study, we developed and characterised human-mouse chimeric monoclonal antibodies targeting the conserved outer membrane protein B (OmpB) of *Rickettsia typhi* as standardised positive control calibrators, rather than as components of a novel diagnostic assay. We evaluated their expression, specificity, and ability to bind native antigen, and assessed their performance as control reagents using the Luminex multiplex platform. By addressing a key bottleneck in rickettsial serodiagnostics, the lack of consistent and renewable positive controls, this work aims to provide a reproducible and standardised solution applicable to assay development, validation, and quality assurance in both research and diagnostic settings.

## Results

### Generation of mouse monoclonal antibodies

**Fig. 1:**
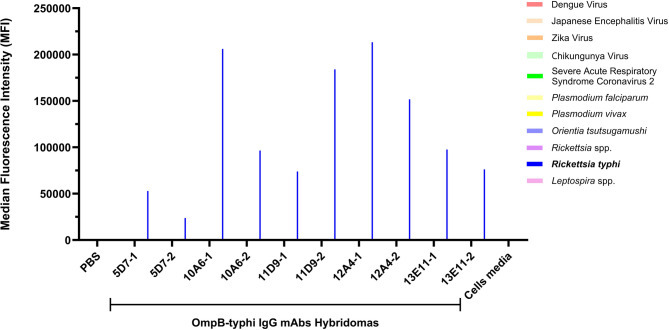
Luminex analysis of mouse IgG mAbs against the OmpB-typhi antigen. Supernatants from 10 hybridoma clones producing mouse IgG mAbs were tested against a febrile disease antigen panel, including the OmpB-typhi antigen, to determine antibody affinity and specificity using a Luminex-based assay. Culture media and PBS were used as negative controls.

Hybridomas producing anti-OmpB-typhi antibodies were successfully generated. Most of the hybridoma clones demonstrated preferential reactivity to OmpB-typhi in the Luminex assay, with no detectable cross-reactivity to unrelated antigens within the tested antigen panel (Fig. 1). The MFI when the mouse monoclonal antibodies reacted with OmpB-typhi antigen ranged from 23,812 to 213,402. Two out of 10 clones showed high reactivity with the OmpB-typhi antigen and reacted slightly with other antigens. No reactivity was observed in negative controls (either PBS or cell media). The most reactive clones that specifically bound the OmpB-typhi antigen were selected for the development of a chimeric antibody.

### Expression of chimeric antibodies in HEK293T cells

**Fig. 2:**
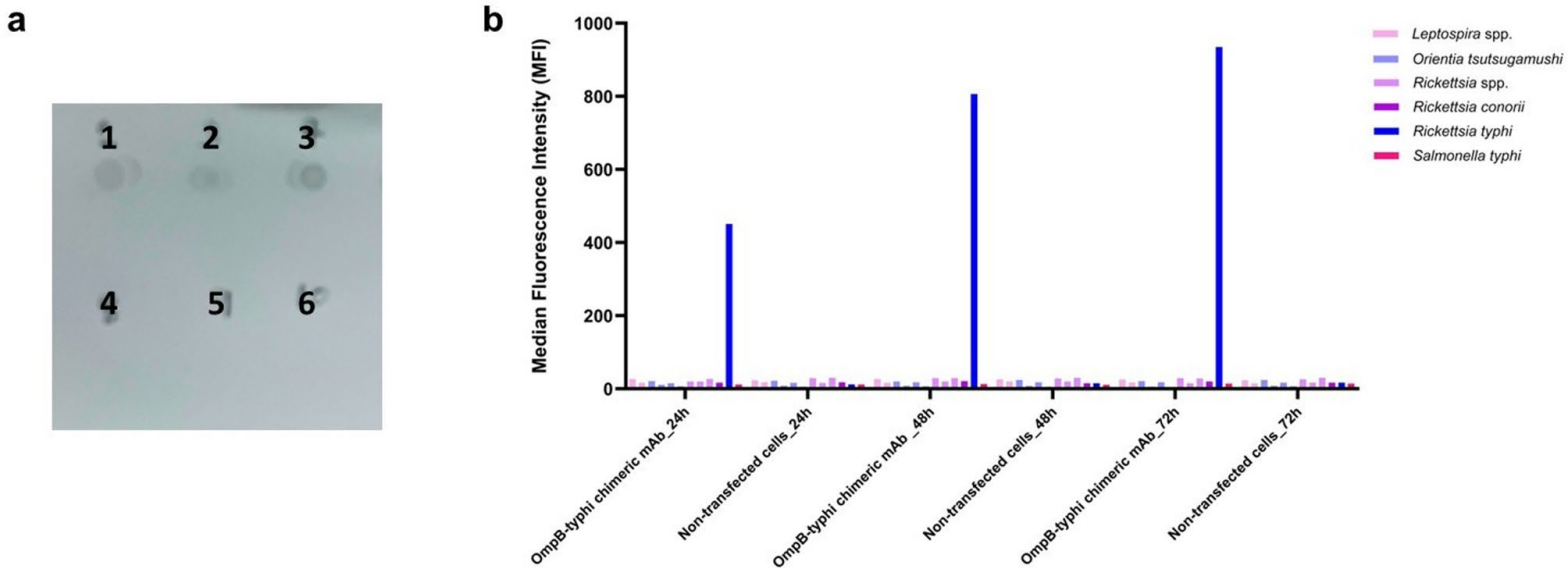
Expression and functional analysis of chimeric antibodies. (**a**) Dot blot analysis of anti-OmpB-typhi chimeric antibodies expression in HEK293T cells. Lanes 1–3 represent supernatants from HEK293T cells co-transfected with anti-OmpB-typhi IgG heavy- and light-chain plasmids; Lanes 4–5 are supernatants from single-plasmid transfected clones; and Lane 6 represents non-transfected cells. (**b**) Supernatants from co-transfected cells containing the anti-OmpB-typhi chimeric antibody (clone 12A4-1) were collected at 24, 48, and 72 h and tested against a febrile disease antigen panel for functional analysis using a Luminex-based assay. Supernatants from non-transfected cells collected at the same time points were used as negative controls.

The anti-OmpB-typhi chimeric antibodies were detected using an anti-human IgG antibody by dot blot analysis. The colourimetric signals were observed as greyish dots in the supernatant samples from HEK293T cells co-transfected with anti-OmpB-typhi IgG heavy- and light-chain plasmids. In contrast, no signal was observed in negative or single-plasmid controls (Fig. 2a). The expression of the representative chimeric antibody derived from hybridoma clone 12A4-1 increased over time (24–72 h), with MFI rising from 451 at 24 h to 935 at 72 h post co-transfection, as measured by the Luminex-based assay. Meanwhile, no fluorescence signal was detected in non-transfected cells, which were used as negative controls (Fig. 2b). Although the chimeric antibody clone 12A4-1 showed good expression and high specificity for the OmpB-typhi antigen, the chimeric antibody derived from hybridoma clone 11D9-2 exhibited MFI values reaching approximately 5,000. Therefore, clone 11D9-2 was selected for subsequent experiments. The reproducibility of the anti-OmpB-typhi chimeric antibody (clone 11D9-2) was evaluated across three independent production batches, with measurements taken in two experimental runs using a Luminex-based assay. Consistent trends in MFI were observed across batches in both runs (Fig. S1 and Table S1). Descriptive statistical analysis demonstrated relatively low within-run variability (CV = 9.35% and 9.52%) and moderate variability across runs for individual batches (CV = 14.31–22.56%), consistent with expected inter-assay variability in multiplex serological assays^15^ (Table S2).

### Chimeric antibodies retain the ability to bind native antigens

**Fig. 3:**
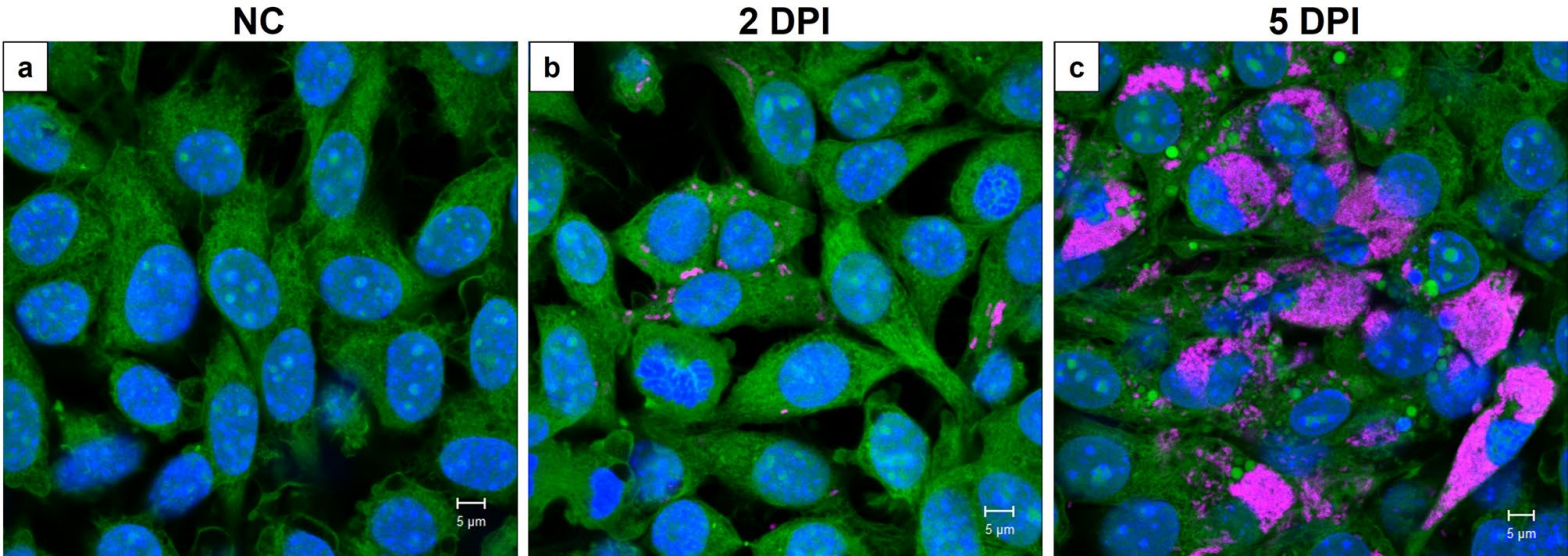
Visualisation of anti-OmpB-typhi chimeric antibody binding to the rickettsial surface. Immunofluorescence microscopic assays were used to demonstrate the specific binding of the chimeric antibody (magenta) to *R. typhi* in L929 cells at 2 DPI (**b**) and 5 DPI (**c**). The infected cells were immobilised on formaldehyde-fixed Ibidi® chamber slides and pre-treated with the anti-OmpB-typhi chimeric antibody. Non-infected cells were used as a negative control (**a**). CellMask™ (green) labels the plasma membrane of host cells, and Hoechst (blue) stains nuclear DNA. Scale bar = 5 µm.

An immunofluorescence microscopy (IFM) assay was performed to examine the binding ability of the anti-OmpB-typhi chimeric antibody (clone 11D9-2) to the outer membrane protein antigen of *R. typhi* in a native antigen context (Fig. 3). Specific immunostaining with the chimeric antibody was observed on the surface of bacteria at 2- and 5-days post-infection (DPI) in L929 cells (Fig. 3b and c). No specific antibody staining was observed in negative control (NC) cells (Fig. 3a).

### Specificity analysis using Luminex multiplex assay

**Fig. 4:**
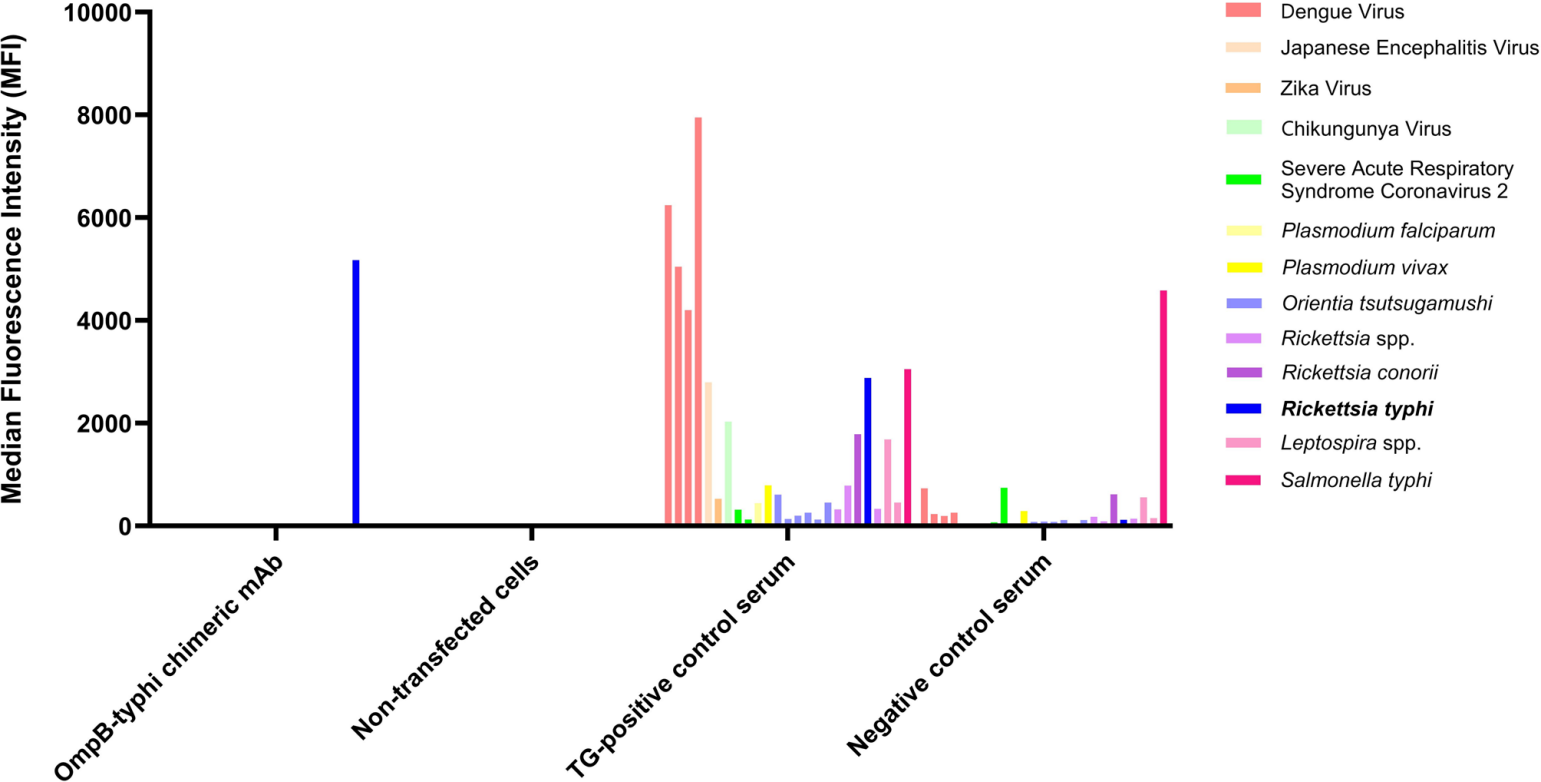
Affinity comparison between anti-OmpB-typhi chimeric antibody and patient-derived TG-positive control serum. The anti-OmpB typhi chimeric antibody (clone 11D9-2) and a positive control serum obtained from a typhus group-infected patient were tested against a febrile disease antigen panel to assess specificity and cross-reactivity using a Luminex assay. Supernatants collected from non-transfected cells and healthy control serum were included as negative controls.

The chimeric antibody (clone 11D9-2) specifically bound the OmpB-typhi antigen (MFI = 5,174) with no detectable cross-reactivity to other rickettsial or non-rickettsial antigens within the evaluated antigen panel (Table 1). In contrast, although the TG-positive control sera showed similar reactivity with OmpB-typhi antigen (MFI = 2,881), they displayed cross-reactivity with multiple antigens, as shown in Fig. 4 and Table 1. No specific reactivity was observed in both the negative control serum and the non-transfected cells’ supernatant.

**Table 1:** Median Fluorescence Intensity (MFI) of reactivity of the OmpB-typhi chimeric mAb with recombinant OmpB-typhi and a febrile illness antigens panel.

Pathogen	Antigen	Antigen ID	Median fluorescence intensity (MFI)			
			OmpB-typhi chimeric mAb	Non-transfected cells	TG-positive control serum	Negative control serum
Dengue virus	Non-structural protein 1	DENV1_NS1	13	15	6241	733
		DENV2_NS1	18	16	5043	232
		DENV3_NS1	16	16	4201	193
		DENV4_NS1	11	14	7947	256
Japanese encephalitis virus	Non-structural protein 1	JEV_NS1	11	11	2796	21
Zika virus	Non-structural protein 1	ZIKVSU_NS1	12	11	530	23
Chikungunya virus	Viral-like particle	CHIKV_VLP	12	13	2028	20
Severe acute respiratory syndrome coronavirus 2	Nucleocapsid protein	SARS-CoV-2_NP	20	19	319	74
	Spike protein	SARS-CoV-2_spike	15	14	124	741
*Plasmodium falciparum*	Merozoite surface protein 1	Pf_MSP1	10	11	445	46
*Plasmodium vivax*		Pv_MSP1	15	15	791	289
*Orientia tsutsugamushi*	56 kDa Type-Specific antigen	OT-TSA56FL-Gilliam	10	9	609	82
		OT-TSA56FL-Kato	15	12	133	86
		OT-TSA56FL-Karp	13	12	201	82
		OT-TSA56FL-TA686	16	17	256	115
		OT-TSA56FL-TA716	14	14	457	115
		OT-TSA56FL-TA763	14	13	127	34
*Rickettsia* spp.	High temperature requirement protease A	HtrA-72	29	30	324	176
	Rickettsia actin-based motility A	RickA	21	24	785	94
	Outer membrane protein A	OmpA-L	20	21	332	140
*R. conorii*	Outer membrane protein B	OmpB-conorii	18	18	1787	617
*R. typhi*		**OmpB-typhi**	**5174**	**15**	**2881**	**118**
*Leptospira* spp.	Outer membrane lipoprotein	LipL32	20	19	457	153
	Leptospiral immunoglobulin-like A protein	LigANI	20	16	1685	555
*Salmonella typhi*	Haemolysin E	HlyE	16	14	3051	4584

Bold indicates the reactivity with *R. typhi* OmpB antigen.

## Discussion

This study demonstrated that human-mouse chimeric mAbs targeting the conserved OmpB antigen of *R. typhi* can serve as promising positive control candidates in serological assays. By combining the variable regions of murine mAbs with the human IgG constant domain, we produced a renewable, specific reagent potentially compatible with widely used serological platforms.

The development of chimeric monoclonal antibodies provides a reproducible and standardised alternative to patient sera as positive controls in serological assays. By targeting conserved epitopes on *Rickettsia* spp. antigens such as OmpB, these chimeric antibodies maintain binding specificity while reducing the batch-to-batch variability associated with human serum controls. Expression in HEK293T cells provides a potentially scalable and reproducible production platform, facilitating long-term reagent availability and supporting applications such as external quality assurance panels, assay lot validation, and proficiency testing.

Unlike convalescent human sera, which are inherently variable and in limited supply, chimeric mAbs may offer improved batch-to-batch consistency, as demonstrated in Tables S1 and S2 and Fig. S1, and eliminate concerns about donor-specific antibody titers or cross-reactivity. The chimeric antibodies demonstrated high apparent specificity and binding to native antigen, as shown in immunofluorescence and Luminex-based assays. Notably, they exhibited no detectable cross-reactivity to non-target antigens within the evaluated panel, potentially addressing an important limitation of current serodiagnostic controls. Previous efforts to use chimeric antibodies for typhus diagnosis have focused mainly on scrub typhus and ELISA platforms^10^. Our work extends this approach to the typhus group rickettsiae and demonstrates its compatibility with multiplex assays. Moreover, the use of HEK293T cells for expression provides a robust and scalable system for mAb production.

Although the chimeric antibody showed reduced MFI in the multiplex assay compared with the parental mouse monoclonal antibody, this reduction might be due to differences in detection platforms between anti-mouse and anti-human antibodies. However, it did not affect the binding affinity of the chimeric antibody, as evidenced by the absence of cross-reactivity with non-target antigens, which may contribute to improved assay consistency across batches. Furthermore, the presence of a human constant region may allow compatibility with existing diagnostic platforms that utilise anti-human secondary antibodies^10,13,16^. This enables recognition by standard anti-human IgG or IgM secondary antibodies commonly used in serological platforms, including ELISA, IFA, and Luminex-based assays, without requiring modification of the detection system.

The three independent production batches of 11D9-2 have demonstrated preliminary batch-to-batch consistency in chimeric monoclonal antibody production, rather than performing a full optimisation or validation of assay performance. Each batch was tested against a multiplex Luminex panel comprising 25 antigens spanning 10 infectious disease groups (as described in the Materials and Methods, Fig. 4, and Table 1). Background reactivity for non-target antigens was consistently observed at MFI values below 50 under the assay conditions used. Across this panel, the chimeric antibody (clone 11D9-2) consistently demonstrated high specificity for OmpB-typhi, with no detectable cross-reactivity, supporting assay specificity and preliminary reproducibility. The two independent assay runs performed on different days further support the reproducibility of performance. However, the technical replicates (e.g., triplicate wells per batch) were not included in this study, which limits formal assessment of intra-assay precision. Future studies should incorporate replicate measurements and expanded statistical analyses to strengthen assay validation further.

The use of chimeric mAbs at a 1:10 dilution in this study represents an initial working concentration intended to demonstrate proof of concept for their application as standardised control reagents. This concentration was not fully optimised, and further refinement may improve assay performance. Future work could therefore focus on enhancing expression yield through codon optimisation and on developing stable expression systems, such as CHO cell lines, to improve scalability and batch-to-batch consistency. In addition, implementing affinity-based purification strategies may improve product purity and reproducibility. Optimisation of formulation conditions, including buffer composition and lyophilisation, will also be important to ensure long-term stability during storage and transport. Collectively, these improvements would support the broader application of the developed chimeric antibodies across serological platforms, particularly in rapid diagnostic tests (RDTs), where reagent stability is essential. Furthermore, evaluation against a wider panel of antigens and related pathogens would strengthen evidence for specificity and robustness.

These findings have significant implications for assay standardisation and regulatory validation. Chimeric mAbs provide a practical alternative to human-derived positive controls, which are often logistically challenging, raise ethical concerns in sourcing large quantities from patients, present immunological variability between individuals, and have limited stability during long-term storage and distribution. As well-defined and scalable reagents, chimeric mAbs offer a reproducible source of reference standards and calibration materials that can be readily shared across laboratories and incorporated into quality assurance programs for regulatory validation across certified diagnostic facilities. Furthermore, their use can enhance the reproducibility of diagnostic kits, supporting more reliable point-of-care testing in endemic regions. In the long term, fully human or bispecific antibodies could further enhance specificity and clinical utility.

This study has several limitations. First, the reproducibility assessment was based on a limited number of independently produced batches and experimental runs, and technical replicate measurements were not included. Second, specificity was evaluated using a predefined antigen panel and may not fully represent potential cross-reactivity against broader rickettsial or non-rickettsial antigen diversity. Third, the chimeric antibodies were evaluated primarily using a Luminex-based platform, and additional validation across other serological platforms, including ELISA and IFA, would strengthen evidence for broader applicability. Finally, long-term stability, storage performance, and inter-laboratory reproducibility were not assessed in this study and should be addressed in future work.

In conclusion, the development of chimeric monoclonal antibodies as positive controls represents a promising approach to a key limitation in rickettsial serodiagnostics. By enabling improved reproducibility, standardisation, and assay comparability, these reagents may support diagnostic development, assay standardisation, and quality assurance efforts, and improved surveillance of rickettsial diseases in both endemic and non-endemic settings.

## Materials and methods

### Ethics declarations

All animal procedures related to antibody production were carried out by GenScript Biotech (Nanjing, China) at the GenScript animal facility in accordance with established institutional animal welfare protocols approved by the GenScript Institutional Animal Care and Use Committee (IACUC; approval number GS-ANT23-011-02-0363). The facility is accredited by the Office of Laboratory Animal Welfare (OLAW; assurance number F16-00214 [A5892-01]) and complies with the Association for Assessment and Accreditation of Laboratory Animal Care (AAALAC) guidelines, the “Guide for the Care and Use of Laboratory Animals”, Public Health Service (PHS) Policy, and relevant Chinese government animal use licenses (SYXK(SU)2023-0016 and SYXK(SU)2023-0017).

### Development of mouse monoclonal antibodies and hybridoma screening

Mouse immunisation and hybridoma generation were performed by a commercial provider (GenScript Biotech, Nanjing, China), where the BALB/c mice were purchased from a certified supplier (Beijing Weitong Lihua Experimental Animal Technology Co., Ltd). In brief, mice were immunised subcutaneously with a recombinant OmpB-typhi protein antigen, previously developed in our laboratory^11^. Booster immunisations were administered at two-week intervals until sufficient serum antibody titers were achieved. Anaesthesia was performed using 1–2% of isoflurane inhalation at a flow rate of 1.5–2.5 L per minute. Mice were euthanised under deep anaesthesia by exsanguination, and the lymphocytes were harvested from the spleen or lymph nodes for hybridoma development. The resulting hybridoma clones were screened for OmpB-typhi-specific antibody production using a Luminex-based assay. Recombinant OmpB-typhi protein and antigens associated with febrile illnesses, including dengue virus serotype 1–4 (DENV1-NS1—DENV4-NS1), Japanese encephalitis virus (JEV-NS1), Zika virus (ZIKVSU-NS1), Chikungunya virus (CHIKV-VLP), *Plasmodium falciparum* (Pf-MSP1) and *Plasmodium vivax* (Pv-MSP1), severe acute respiratory syndrome coronavirus 2 (SARS-CoV2-NP and SARS-CoV2-spike), three strains of *Orientia tsutsugamushi* (OT-TSA56-Gilliam, Kato, and Karp), *Rickettsia typhi* (OmpB-typhi), *Rickettsia* spp. (RickA) and pathogenic *Leptospira* spp. (LipL32 and LigANI) were individually coupled to magnetic microsphere beads (Luminex MagPlex™, USA). The concentration of each antigen was optimised for its assigned bead region. Coupled beads were pooled, and 50 µL of the bead mixture was transferred to each well of a 96-well flat-bottom plate (Bio-Rad, USA). The hybridoma supernatants containing monoclonal antibodies (mAbs; n = 10) were diluted 1:100 and added in equal volumes to each well containing the bead mixture. Culture media and phosphate-buffered saline (PBS) were included as negative controls. The plate was incubated at room temperature for 30 min with shaking at 600 rpm. Following incubation, the beads were washed three times with 100 µL of PBS containing 0.05% Tween-20. Subsequently, 50 µL of phycoerythrin (PE)-conjugated anti-mouse IgG H&L secondary antibody (Abcam, UK) was added at a 1:100 dilution to each well, and the plate was incubated at room temperature in the dark for 15 min with shaking at 600 rpm. The plate was rewashed three times before adding 100 µL of PBS containing 1% bovine serum albumin (BSA) and 0.05% Tween-20, and incubated in the dark for 5 min at 600 rpm. After that, the median fluorescence intensity (MFI) was measured using a Luminex multiplex platform (xMAP INTELLIFLEX®, Luminex Corp., USA). Hybridoma supernatants exhibiting strong fluorescence reactivity were selected for expansion and monoclonal antibody production. Sequencing of the mouse antibody variable domains, including light (Vκ) and heavy (V_H_) chains, from selected hybridomas, was performed by a commercial service provider (GenScript Biotech, China).

### Construction of human-mouse chimeric antibodies

Selected hybridoma clones (12A4-1 and 11D9-2) were propagated, and total RNA was isolated using RNeasy kits (Qiagen, Germany). The isolated RNA was reverse-transcribed into cDNA using the SuperScript™ III First-Strand Synthesis System (Thermo Fisher Scientific, USA). The variable regions of mouse anti-OmpB-typhi IgG monoclonal antibodies were amplified by PCR using specific primers containing restriction enzyme sites including EcoRI, NheI and BsiWI (Table 2). The primers were designed based on the nucleotide sequences of the antibody variable domains. The PCR products were cloned into expression vectors containing the human IgG constant regions of the light chain (pFUSE2ss-CLIg-hk) and heavy chain (pFUSEss-CHIg-hG1). Recombinant plasmids carrying mouse variable regions fused to human IgG constant regions were co-transfected into HEK293T cells using Lipofectamine™ 3000 Transfection Reagent (Invitrogen™, Thermo Fisher Scientific, USA), following the manufacturer’s protocol.

**Table 2:** Primers for amplification of anti-OmpB-typhi IgG variable regions.

No	Variable regions	Primer name	Primer sequence (5'-3')	Size (bp)
1	Heavy chain	OBTVH12A4F	TAAGCAGAATTCGCAGGTCCAACTGCAGCAGCC	357
2		OBTVH12A4R	TGCTTAGCTAGCTGAGGAGACGGTGACTGAGG	
3		OBTVH11D9F	TAAGCAGAATTCGGAATTTCAGCTCCAGCAGTCT	336
4		OBTVH11D9R	TGCTTAGCTAGCTGAGGAGACTGTGAGAGTGGT	
5	Light (kappa) chain	OBTVL12A4F	TAAGCAGAATTCAGACATTGTGATGTCACAGTC	339
6		OBTVL12A4R	TGCTTACGTACGTTTCAGCTCCAGCTTGGTC	
7		OBTVL11D9F	TAAGCAGAATTCAGACATCAAGATGACCCAGT	321
8		OBTVL11D9R	TGCTTACGTACGTTTTATTTCCAGCTTGGTCCC	

Underlined sections in primer sequences indicate the restriction enzyme sites.

### Expression and detection of chimeric antibodies

Culture supernatants were collected at 24, 48, and 72 h post-transfection. Expression of anti-OmpB-typhi chimeric antibodies was confirmed using dot blot analysis. Briefly, 10 µL of each supernatant was spotted onto a nitrocellulose membrane and dried at 37 °C in a HybEZ™ II Oven (ACD, USA). The membrane was blocked for 1 h at room temperature with blocking buffer consisting of Tris-buffered saline (TBS) containing 3% BSA and 0.1% Tween-20 under gentle orbital shaking. The membrane was washed 3 times with TBS containing 0.1% Tween-20 (TBST) for 5 min per wash, then incubated for 1 h with horseradish peroxidase (HRP)-conjugated goat anti-human IgG cross-adsorbed secondary antibody (Thermo Fisher Scientific, USA) at a 1:1,000 dilution. After 3 washes, the membrane was equilibrated with PBS. CN/DAB substrate was added, and the mixture was incubated until a colourimetric signal developed. The reaction was stopped by rinsing the membrane with distilled water. Supernatants from non-transfected cells and vector-only-transfected cells served as negative controls.

### Evaluation of native antigen binding ability

The ability of the anti-OmpB-typhi chimeric antibody to bind the native antigen, outer membrane protein B (OmpB) of *Rickettsia typhi* (*R. typhi*), was evaluated using an immunofluorescence microscopic (IFM) assay. L929 cells were seeded onto a chamber slide (Ibidi®, Germany) and incubated at 37 °C with 5% CO_2_ until they reached confluence. The cells were then infected with *R. typhi* at a multiplicity of infection (MOI) of 20 and incubated at 37 °C with 5% CO_2_ for 2 and 5 days. Non-infected cells were used as a negative control (NC). At the indicated time points, both infected and non-infected cells were fixed with 4% formaldehyde at room temperature for 10 min, followed by 3 washes with PBS. The formaldehyde-fixed cells were incubated with absolute ethanol at 4 °C for 1 h. After washing, cells were permeabilised by incubation with PBS containing 0.5% TritonX-100 at 4 °C for 30 min. The cells were then blocked with blocking buffer (PBS containing 3% BSA and 0.1% Tween-20) at room temperature for 1 h. After removal of the blocking buffer, the cells were incubated overnight at 4 °C with the anti-OmpB-typhi chimeric antibody at a dilution of 1:10. Subsequently, secondary antibody and counterstains were added, including anti-human IgG 640 (Sigma-Aldrich, USA) (1:1,000), Hoechst 33,342 nucleic acid stain (Invitrogen, USA) (1:2,000) and CellMask™ plasma membrane stain (Invitrogen, USA) (1:10,000). The cells were incubated at 37 °C for 1 h in the dark. After incubation, cells were washed three times with PBS and mounted with mounting medium. The chamber slides were sealed with coverslips and stored at 4 °C in the dark until visualisation with a confocal microscope.

### Functional validation via Luminex assay

The specificity and binding activity of the anti-OmpB-typhi chimeric antibody were validated using a Luminex multiplex platform (xMAP INTELLIFLEX®, Luminex Corp., USA) containing a panel of rickettsial and non-rickettsial antigens. Antigen-coupled beads were mixed with the chimeric antibody and positive or negative control samples at a 1:1 ratio in a 96-well, flat-bottom plate (Bio-Rad, USA). The chimeric antibody and serum samples were diluted to 1:10 and 1:100, respectively, prior to incubation with the beads. The plate was incubated at room temperature for 30 min with shaking at 600 rpm. After 3 washes with PBS containing 0.05% Tween-20, 50 µL of PE-conjugated anti-human IgG secondary antibody (Abcam, UK) was added to each well at a 1:100 dilution. The plate was incubated for 15 min at room temperature in the dark with shaking at 600 rpm. The plate was then rewashed before adding 100 µL PBS containing 1% BSA and 0.05% Tween-20. After incubation for 5 min at room temperature in the dark with shaking at 600 rpm, the MFI was measured using the Luminex multiplex platform. Data analysis and graph generation were performed using GraphPad Prism software (version 10.0) (GraphPad Software, Boston, Massachusetts, USA, 2023).

Comparative analyses were performed using Typhus group (TG)-positive control sera from confirmed patient samples. The supernatant from non-transfected cell cultures and the serum from a non-infected individual were used as negative controls.

## Supplementary Information

Supplementary Information.

## Data Availability

The datasets generated during the current study are available from the corresponding author upon reasonable request.
