# Supplementary material for: Development of chimeric monoclonal antibodies as standardised positive controls in rickettsial serodiagnosis
Source: Sci Rep. 2026 May 29;16:24958. doi: 10.1038/s41598-026-55470-4 (PMC13462989; doi:10.1038/s41598-026-55470-4)
Supplement: Supplementary file 1 [file 41598_2026_55470_MOESM1_ESM.docx]

**Supplementary Information**

**Table S1.** Comparison of independent production batches across two experimental runs to assess reproducibility of the anti–OmpB-typhi chimeric antibody (clone 11D9-2).

| Pathogen | Antigen ID | Median Fluorescence Intensity (MFI) | | | | | |
| --- | --- | --- | --- | --- | --- | --- | --- |
|  |  | **Run 1** | | | **Run 2** | | |
|  |  | Batch 1 | Batch 2 | Batch 3 | Batch 1 | Batch 2 | Batch 3 |
| Dengue virus | DENV1_NS1 | 14 | 9 | 12 | 14 | 13 | 14 |
|  | DENV2_NS1 | 16 | 15 | 18 | 22 | 18 | 20 |
|  | DENV3_NS1 | 15 | 15 | 16 | 16 | 16 | 17 |
|  | DENV4_NS1 | 9 | 11 | 12 | 13 | 11 | 13 |
| Japanese encephalitis virus | JEV_NS1 | 11 | 11 | 10 | 11 | 11 | 8 |
| Zika virus | ZIKVSU_NS1 | 12 | 15 | 10 | 15 | 12 | 11 |
| Chikungunya virus | CHIKV_VLP | 13 | 11 | 14 | 15 | 12 | 14 |
| Severe acute respiratory syndrome coronavirus 2 | SAR-CoV-2_NP | 19 | 21 | 19 | 18 | 20 | 18 |
|  | SAR-CoV-2_spike | 15 | 15 | 19 | 18 | 15 | 19 |
| *Plasmodium falciparum* | Pf_MSP1 | 9 | 12 | 10 | 11 | 10 | 10 |
| *Plasmodium vivax* | Pv_MSP1 | 15 | 15 | 13 | 17 | 15 | 18 |
| *Orientia tsutsugamushi* | OT-TSA56FL-Gilliam | 12 | 9 | 10 | 8 | 10 | 9 |
|  | OT-TSA56FL-Kato | 15 | 11 | 13 | 13 | 15 | 10 |
|  | OT-TSA56FL-Karp | 14 | 11 | 16 | 13 | 13 | 12 |
|  | OT-TSA56FL-TA686 | 15 | 14 | 17 | 14 | 16 | 14 |
|  | OT-TSA56FL-TA716 | 16 | 16 | 16 | 13 | 14 | 15 |
|  | OT-TSA56FL-TA763 | 13 | 13 | 15 | 13 | 14 | 13 |
| *Rickettsia* spp. | HtrA-72 | 31 | 31 | 36 | 28 | 29 | 33 |
|  | RickA | 21 | 22 | 21 | 24 | 21 | 24 |
|  | OmpA-L | 19 | 20 | 17 | 20 | 20 | 19 |
| *R. conorii* | OmpB-conorii | 16 | 17 | 18 | 15 | 18 | 14 |
| *R. typhi* | **OmpB-typhi** | **3219** | **3884** | **3571** | **4440** | **5174** | **4375** |
| *Leptospira* spp. | LipL32 | 19 | 18 | 21 | 20 | 20 | 18 |
|  | LigANI | 17 | 17 | 17 | 17 | 20 | 17 |
| *Salmonella typhi* | HlyE | 14 | 15 | 15 | 14 | 16 | 13 |

**Bold** indicates the reactivity with *R. typhi* OmpB antigen

**Table S2.** Summary statistics for preliminary reproducibility assessment of the anti–OmpB-typhi chimeric antibody (clone 11D9-2).

| **Analysis level** | **Group** | **Reported MFI values** | **Mean MFI** | **SD** | **%CV** |
| --- | --- | --- | --- | --- | --- |
| Within run | Run 1 | 3219, 3884, 3571 | 3558.3 | 332.7 | 9.35 |
| Within run | Run 2 | 4440, 5174, 4375 | 4663.0 | 443.8 | 9.52 |
| Across run | Batch 1 | 3219, 4440 | 3829.5 | 863.8 | 22.56 |
| Across run | Batch 2 | 3884, 5174 | 4529.0 | 912.2 | 20.14 |
| Across run | Batch 3 | 3571, 4375 | 3973.0 | 568.4 | 14.30 |

**Figure S1.** Reproducibility assessment of the anti–OmpB-typhi chimeric antibody (clone 11D9-2) across independent production batches and experimental runs.

Median fluorescence intensity (MFI) values obtained from three independently produced batches of the anti–OmpB-typhi chimeric antibody (clone 11D9-2) evaluated in two separate Luminex assay runs. Consistent reactivity against the OmpB-typhi antigen was observed across batches and experimental runs, supporting preliminary batch-to-batch consistency and assay reproducibility.
